# Supplementary material for: Clinical Effectiveness and Pharmacokinetics of Dalbavancin in Treatment-Experienced Patients with Skin, Osteoarticular, or Vascular Infections
Source: Pharmaceutics. 2022 Sep 6;14(9):1882. doi: 10.3390/pharmaceutics14091882 (PMC9501058; doi:10.3390/pharmaceutics14091882)
Supplement: Supplementary file 1 [file pharmaceutics-14-01882-s001.zip › pharmaceutics-1782826-supplementary.pdf]

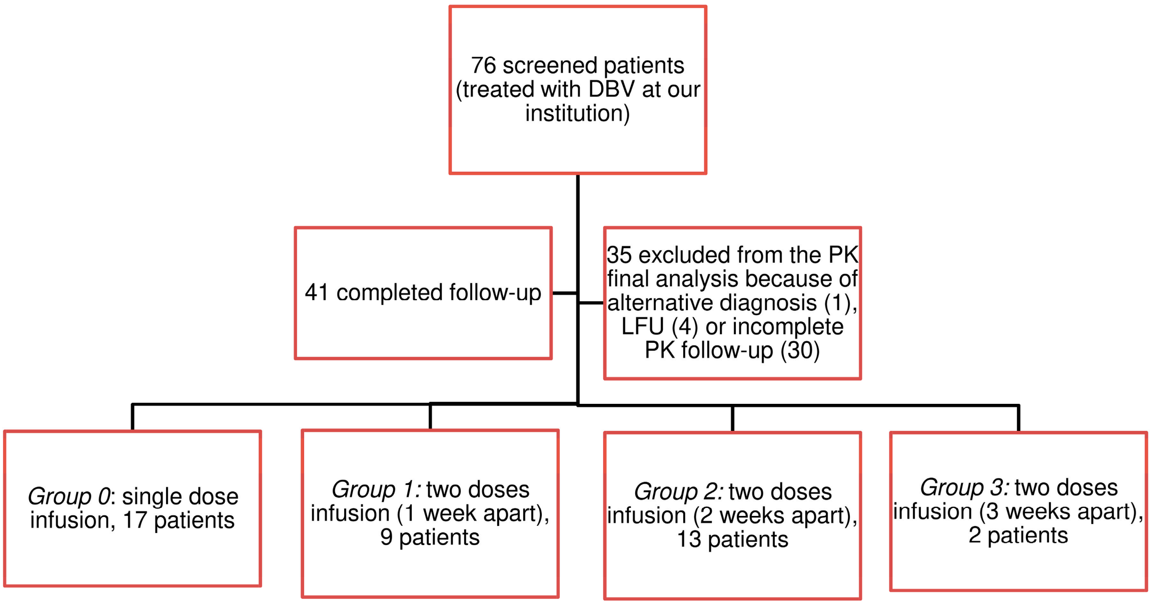

Figure S1. Flow in the selection of patients from screening to complete follow-up. DBV: dalbavancin; LFU: lost to follow-up; PK: pharmacokinetic.

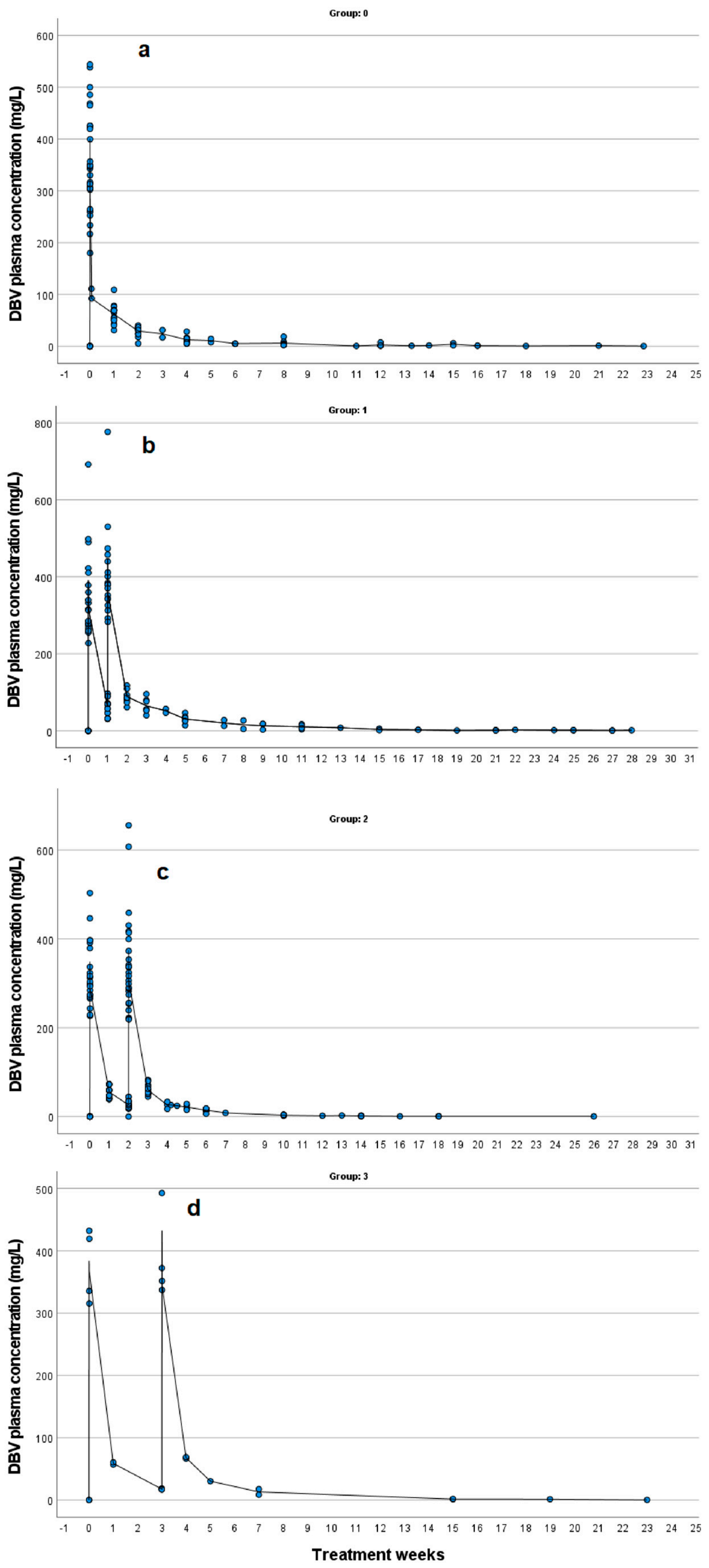

Figure S2. Mean PK profiles in each treatment group. Panel (a) group 0; Panel (b) Group 1; Panel (c) group 2; Panel (d) group 3. X axis = time from the first dose (weeks).
